# Large Choroidal Melanocytoma Simulating Choroidal Melanoma: A Difficult Differential Diagnosis and an Inevitable Enucleation

**DOI:** 10.1155/2020/8890857

**Published:** 2020-11-21

**Authors:** Taoufik Abdellaoui, Soukaina Belfaiza, Yassine Malek, Mohamed-amine Essaoudi, Fouad Elasri, Karim Reda, Abdelbarre Oubaaz

**Affiliations:** ^1^Department of Ophthalmology, Military Teaching Hospital Med-V, Rabat, Morocco; ^2^Department of Pathology, Military Teaching Hospital Med-V, Rabat, Morocco

## Abstract

**Purpose:**

To describe a case of choroidal melanocytoma mimicking a melanoma.

**Methods:**

Retrospective case report. *Patient*. A 48-year-old Moroccan woman presented with progressive, painless decreased vision in her left eye for 2 months.

**Results:**

Her visual acuity was light perception in the left eye and 20/20 in the right one. Fundus examination and fluorescein angiography of the left eye showed a total retinal detachment with a large superior brownish mass. The clinical examination, B-scan ultrasonography, and magnetic resonance imaging all suggested a malignant melanoma. Consequently, the eye was enucleated. The histopathology later revealed a benign melanocytoma of the choroid. *Discussion*. Melanocytoma is a rare benign pigmented tumor. It is classically described as a tumor of the optic nerve head, but there are some exceptional case reports of uveal tract locations (iris, ciliary body, and choroid). In such cases, it can be difficult to clinically differentiate a melanocytoma from a malignant melanoma.

## 1. Introduction

Melanocytoma is a rare pigmented primary benign tumor that is usually located in the optic disc and the anterior portion of the optic nerve [1] where the clinical diagnosis is relatively easy. However, in very rare instances, it can be localized in any part of the uveal tract (iris, ciliary body, and choroid) [2–4]. We report the case of a 48-year-old woman with a large choroidal lesion of the left eye. The clinical examination, B-scan ultrasonography, and magnetic resonance imaging all suggested a malignant melanoma. Consequently, the eye was enucleated. The histopathology later revealed a benign melanocytoma of the choroid.

## 2. Case Report

**Figure 1:**
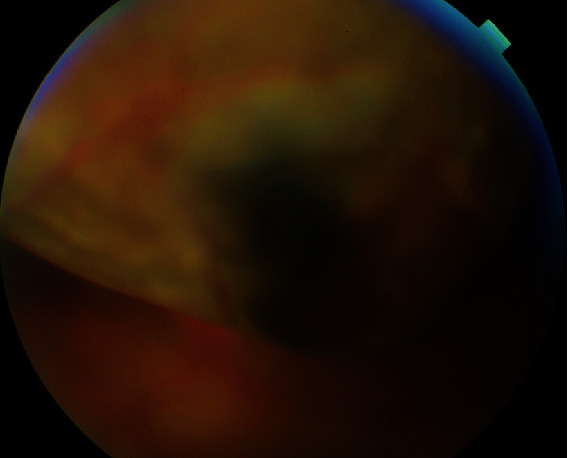
Deeply pigmented choroidal lesion with a retinal detachment.

A 48-year-old Moroccan woman presented with progressive, painless decreased vision in the left eye for 2 months. Her visual acuity was light perception in the left eye and 20/20 in the right one. Biomicroscopy of the left eye showed no conjunctival hyperemia, a clear cornea, and no inflammation in the anterior chamber. Intraocular pressure was normal. Fundus examination and fluorescein angiography of the left eye showed a retinal detachment (RD) with a large superior brownish mass (Figure 1). The right eye examination was unremarkable.

**Figure 2:**
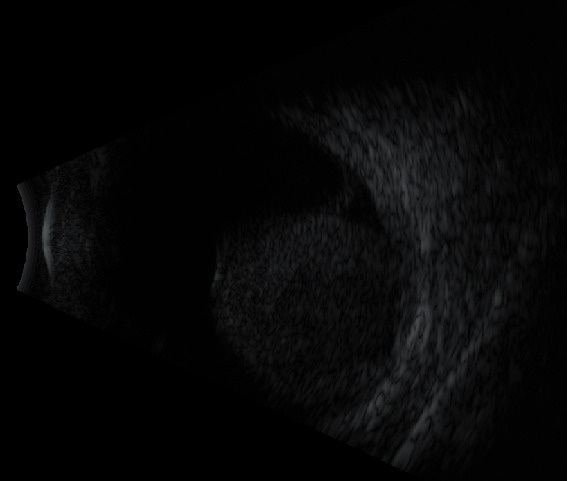
B-scan ultrasonography: large choroidal dome-shaped mass, with neither acoustic hollowing nor calcification, associated with a retinal detachment (subretinal fluid around the mass, without RD extending to the periphery).

**Figure 3:**
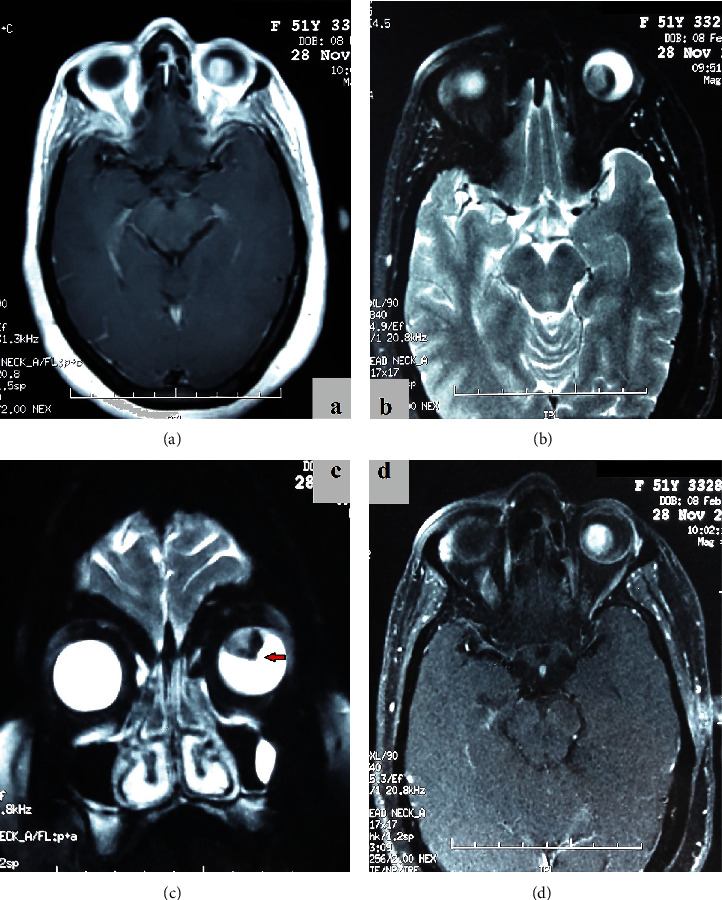
MRI of the orbits: (a) tumor showing high signal intensity on T1-weighted images; (b) heterogeneous appearance and low signal intensity on T2-weighted images; (c) T2-weighted images on a coronal plane: heterogeneous appearance and low intensity with a collar-button extension (arrow); (d) tumor shows enhancement on T1-weighted images with fat suppression, after Gadolinium administration.

B-scan ultrasonography of the left eye revealed a large choroidal dome-shaped mass, surrounded by subretinal fluid, with neither acoustic hollowing nor calcification (Figure 2). MRI of the orbits showed an intraocular tumor measuring 12 × 11 × 11mm, which displayed a high signal intensity on T1-weighted images, low signal intensity on T2, with a collar-button extension, and an enhancement on T1-weighted images after Gadolinium administration. This appearance was consistent with a choroidal melanoma. There was a scleral thickening and no evidence of extraocular tumor extension (Figure 3). Thoracoabdominal CT scan did not reveal any sign of metastasis. According to the results, this intraocular tumor was highly suggestive of a choroidal melanoma; an enucleation of the left eye was therefore indicated, with the patient's consent.

**Figure 4:**
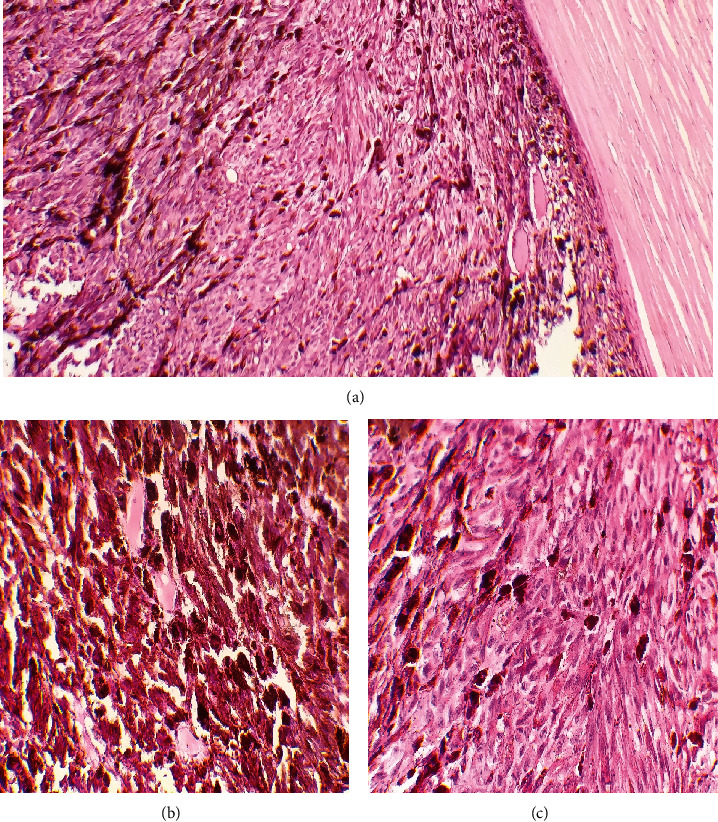
Pathologic findings: (a) pigmented tumor composed of fusiform cells with brownish cytoplasm (hematoxylin-eosin stain, original magnification 10x). Two different zones: (b) the first one is highly pigmented with brownish cytoplasm, and (c) the second zone is less pigmented where we can see the nuclear details: fusiform cells with elongated nuclei and small nucleoli, without necrosis, atypia, or mitotic activity (hematoxylin-eosin stain, original magnification 40x).

The histopathological study of the enucleated eye showed a brownish-black pigmented tumor arising from the choroid, composed of fusiform cells with brownish cytoplasm, elongated nuclei, and small nucleoli. No signs of necrosis or malignancy were found (Figure 4). Immunohistochemical analysis demonstrated a positive cytoplasmic reaction for S-100 protein and HMB-45 monoclonal antibody. These findings were consistent with a choroidal melanocytoma, not a melanoma.

## 3. Discussion

Melanocytoma is a distinctive variant of melanocytic nevus. It is a rare benign pigmented tumor. It is classically described as a tumor of the optic nerve head, but there are some exceptional case reports of uveal tract locations [2–4]. In such cases, it can be difficult to clinically differentiate a melanocytoma from a malignant melanoma. In spite of it being benign, melanocytomas can undergo malignant changes [5]. Regarding the size, melanocytomas rarely exceed 2 disc diameter in size [6]; however, our patient presented an unusually large tumor.

As opposed to the optic disc melanocytoma, the extrapapillary uveal melanocytoma is generally more challenging to diagnose clinically, since it is similar to other uveal nevi and small uveal malignant melanoma, even more so in case of a choroidal location.

Exudative retinal detachment is the most common abnormality associated with malignant uveal melanoma and is present in up to 75% of eyes bearing this tumor. However, this finding is nonspecific and could be present in both benign and malignant tumors. In a series of 115 eyes with optic disc melanocytoma, Shields et al. found subretinal fluid adjacent to the tumor in 14% of patients [7]. In our patient, the subretinal fluid was limited to the periphery of the tumor. We think that this retinal detachment was due to the mechanical effect of the large tumor rather than being exudative.

In general, it is believed that significant growth of a melanocytic uveal lesion is indicative of a malignant transformation [8]. In our case, considering the large dimensions of the tumor, the more accurate diagnosis seemed to be a melanoma.

In fluorescein angiography, a large choroidal melanocytoma can be extremely difficult to differentiate from a melanoma considering that they both show hyperfluorescence [9]. On B-scan ultrasonography, melanocytomas have been described to have high levels of internal reflectivity. However, this not a characteristic feature. Like our case, some authors have reported some choroidal melanocytomas with relatively low internal reflectivity [9, 10], making the differentiation from melanoma virtually impossible. Magnetic resonance imaging is not superior to ultrasound in diagnosis of choroidal melanoma and differentiation from other lesions [8, 11]. Fine-needle aspiration biopsy is often mentioned as a method of differentiating melanoma from other pigmented tumors. The cytology study is significant if it reveals melanoma cells. Otherwise, if it only shows melanocytoma cells, it is possible that a sampling error has occurred and foci of melanoma cells could have been missed. Tumor endobiopsy of choroidal lesions using a transvitreal 25- or 27-gauge pars plana vitrectomy (PPV) approach offers potential advantages over fine-needle aspiration including direct visualization, providing a large sample adequate for histopathological examination and cytogenetic analysis, as well as lower risk for iatrogenic retinal detachment, vitreous hemorrhage, and seeding of tumor cells to the vitreous body [12, 13]. Abi-Ayad el al. reported 9 choroidal tumors biopsied with 25-gauge vitrectomy system. Biopsies established the diagnosis of uveal melanoma in all cases, with no systemic spread or any intraocular dissemination over a median of 24 months of follow-up [14]. However, in case of a strong clinical presumption of melanoma, like our case, the patients often eventually end up with a drastic procedure, such as enucleation.

## 4. Conclusion

Though rare, choroidal melanocytomas should be considered as a differential diagnosis of uveal tract tumors. A conservative approach can have a sight-saving effect and allow a better quality of life for the patient. However, in some cases, like ours, the clinical appearance with retinal detachment, the size of the tumor, and ultrasound and radiological features did not suggest a melanocytoma. Therefore, enucleation could not have been avoided.

## Data Availability

The data used to support the findings of this study are available from the corresponding author upon request.
